# An Enquiry into Nurse-to-Nurse Collaboration Within the Older People Care Chain as Part of the Integrated Care: A Qualitative Study

**DOI:** 10.5334/ijic.2418

**Published:** 2017-03-31

**Authors:** Terhi Lemetti, Päivi Voutilainen, Minna Stolt, Sini Eloranta, Riitta Suhonen

**Affiliations:** 1 Department of Nursing Science, University of Turku, Helsinki University Hospital, Helsinki, FI; 2 Ministry of Social Affairs and Health, Helsinki, FI; 3 Department of Nursing Science, University of Turku, Turku, FI; 4 University of Turku, Turku, FI; 5 Department of Nursing Science, University of Turku/Turku University Hospital, City of Turku, Welfare Division, Turku, FI

**Keywords:** collaboration, hospital, nurses, integrated care, older people, primary healthcare

## Abstract

**Introduction:**

Health care systems for older people are becoming more complex and care for older people, in the transition between hospital and primary healthcare requires more systematic collaboration between nurses. This study describes nurses’ perceptions of their collaboration when working between hospital and primary healthcare within the older people care chain.

**Theory and methods:**

Using a qualitative approach, informed by grounded theory, six focus groups were conducted with a purposive sample of registered nurses (n = 28) from hospitals (n = 14) and primary healthcare (n = 14) during 2013. The data were analyzed using dimensional analysis.

**Findings:**

Four dimensions of collaboration were identified: 1) Context and Situation, 2) Conditions, 3) Processes and Interactions and 4) The Consequences of nurse-to-nurse collaboration within the older people care chain. These four dimensions were then conceptualized into a model of nurse-to-nurse collaboration.

**Discussion and conclusion:**

Improved collaboration is useful for the safe, timely and controlled transfer of older people between hospital and primary healthcare organizations and also in healthcare education. The findings in this study of nurse-to-nurse collaboration provides direction and opportunities to improve collaboration and subsequently, the continuity and integration in older people care in the transition between organizations.

## Introduction and background

The continuity of care within the older people care chain requires collaboration between different healthcare groups [1] to provide a seamless service, enabling older people to live independently for longer [2,3]. Collaboration between nurses in the care of older people is important in Finland because it is estimated that in 2012 there were over 59,000 nurses working in Finnish healthcare organizations. Of these 59,000, 45.4% worked in hospitals and 19.9% in primary healthcare [4]. The number of older people, often defined as those over 65 [5], is set to increase in Europe over the next 40 years [6] and in Finland was over 1 million in 2012, equating to 18.8% of the total population [7]. Of these older people over 140,000 were frequent users of social and healthcare services [8] and used 90% of the Finnish primary healthcare service provision in 2014 [9]. This service usage by older people is likely to increase as Finland, like many countries in Europe, move towards home-based care [10]. This position is mirrored worldwide with over 19 million nurses [11] working together to integrate care between different parts of various organizations [12] and to promote, maintain and restore the health [13] of over 500 million people aged 60 and over [14].

In Finland, the response to these demographic developments has been the reform of social and health care within a regional government structure [15]. This reform is one of the largest administrative and operational overhauls ever undertaken in Finland [16] making it a legal requirement under the Health Care Act [17] and the Care Services for Older People Act [18], to deliver patient-centered, seamless, integrated care services [19]. One aspect of integrated service provision is the development of care chains in which the movement of patients within an organization and the transfer to other organizations is carefully managed [16]. Developing care chains is an important way of improving the continuity of care within fragmented health care systems [20]. Within the Finnish healthcare chain, the aim is to support independent living and care close to home for as long as possible [21]. Integrated services for older people therefore include the provision of services which are seamless in the transition from one organization to another and demonstrate a clear transfer of responsibilities between health professionals in the care chain [2]. This integration of services impacts on the jobs of hundreds of thousands of people and affects the health services of every citizen in Finland [16]. One of the reasons for the impact of integration is that it requires an increase in collaboration between hospital and primary healthcare nurses. In this context, an increased knowledge and understanding of the nature of collaboration between healthcare professionals is important.

Collaboration has been described as a partnership in power where mutually dependent individuals share decision-making, philosophies and values, information and plans [22]. Collaborating individuals think of themselves as team members who work to achieve a common goal [23] sharing their expertise and responsibilities in a non-hierarchical relationship [23]. A requirement of this process is that the individuals meet, develop relationships and communicate effectively with each other in mutual trust [24].

Staff in hospitals and the primary healthcare sector play a significant role in health care systems worldwide [25] which includes collaborating as part of the management of healthcare between settings [26]. Nurses are essential actors [27] in this collaboration [1] as they provide bridging roles when patients move between organizations [1]. Professionally, nurses are encouraged to strengthen collaborative practices [27,28,29,30] and partnerships [31] though currently, professional collaboration may not be as effective as it could be [32,33]. This possible deficit is especially important at times when organizations are trying to improve organization integration and the continuity of care [34].

The improvement of continuity in care requires an increase in collaboration between nurses especially those working in different organizations. This collaboration works well when there are open communication channels between organizations [32,35] and the effective transfer of information for example, about patients’ medication and treatments [34,35,36,37,38]. Although the lack of research in this area is widely reported [39,40,41,42], McKenna et al. [41] has suggested that 68% of primary healthcare nurses found communication with hospital staff unsatisfactory suggesting poor collaboration. It has been reported that collaboration can be improved by using liaison nurses to organize communication and discharge planning between organizations [43,44,45] further suggesting that collaboration between practicing nurses requires development.

The elements of good collaboration include respect [35,46], trust, openness [46] and an awareness of participating nurses’ roles and responsibilities [37,46,47]. Nursing collaboration could be improved through education, face-to-face meetings and time spent together [36,46]. Collaboration can also be improved through the development of shared objectives between the collaborators [43], job rotation [32] and the use of communication tools such as picture messaging [35,46,48].

Collaboration requires skills [36,37] in decision-making, problem-solving, leadership and conflict management [36,47] and these skills include the ability to acknowledge one’s own humanity and limitations [38]. Nurses seem to have some knowledge and understanding of collaboration, but are unsure how to implement these in clinical practice [40].

This current study describes the nature of nurse-to-nurse collaboration focusing on social processes as nurses work to transfer older people from hospital into primary health care. The study augments the collaboration literature, assisting in the future development of collaboration within the older people care chain.

## Methods

### Aim

To identify a basis for a theory of collaboration through descriptions of nurses’ perceptions of the collaboration between hospital and primary healthcare nurses in the older people care chain.

### Design

This study used a qualitative design informed by grounded theory [49,50,51] and focus groups [52].

### Participants

A purposive sample of 28 registered nurses from hospital (n = 14) and primary healthcare units (n = 14) in one hospital district in Southern Finland was used (Table 1). The hospital-based participants worked in outpatient clinics in surgery, cardiology and an infectious diseases unit. Primary healthcare participants were nurses from home care units. To be included in the study the participants had to (i) be Finnish-speaking registered nurses, (ii) have at least three years nursing experience in a hospital or primary healthcare setting, (iii) be clinically based, caring for older people on a daily basis and (iv) collaborate with hospital or primary healthcare nurses on at least a weekly basis.

**Table 1:** Presentation and demographic description of the participants.

Interview	Organization	Pilot n = 4 Main study n = 28	Participant	Demographic description of the participants

Pilot Focus group	Hospital	4	Nurses, Surgical unit	**Gender:** Female n = 4, Male n = 0 **Age:** 20–29 n = 1 30–39 n = 0 40–49 n = 1 50–59 n = 2 over 60 n = 0
Focus group 1	Hospital	6	Nurses, Infectious diseases unit	**Gender:** Female n = 5, Male n = 1 **Age:** 20–29 n = 0 30–39 n = 1 40–49 n = 1 50–59 n = 4 over 60 n = 0
Focus group 2	Primary healthcare	6	Nurses, Home care	**Gender:** Female n = 6, Male n = 0 **Age:** 20–29 n = 0 30–39 n = 3 40–49 n = 1 50–59 n = 1 over 60 n = 0 (? n = 1)
Focus group 3	Hospital	4	Nurses, Surgical unit	**Gender:** Female n = 4, Male n = 0 **Age:** 20–29 n = 0 30–39 n = 1 40–49 n = 1 50–59 n = 2 over 60 n = 0
Focus group 4	Primary healthcare	2	Nurses, Home care	**Gender:** Female n = 2, Male n = 0 **Age:** 20–29 n = 0 30–39 n = 0 40–49 n = 2 50–59 n = 0 over 60 n = 0
Focus group 5	Primary healthcare	6	Nurses, Home care	**Gender:** Female n = 6, Male n = 0 **Age:** 20–29 n = 0 30–39 n = 2 40–49 n = 1 50–59 n = 2 over 60 n = 1
Focus group 6	Hospital	4	Nurses, Cardiology unit	**Gender:** Female n = 4, Male n = 0 **Age:** 20–29 n = 0 30–39 n = 2 40–49 n = 2 50–59 n = 0 over 60 n = 0

Participants were recruited with the help of the head nurse from the chosen hospitals and primary healthcare units. The first author informed the staff about the study and potential participants received an information letter asking them to inform the head nurse if they were willing to participate. The aim was to recruit at least 20 participants [53] based on reaching a critical mass of data [50].

### Data collection

Data were collected using focus groups (first author) between May and November 2013. The interview guide was tested in a pilot study (n = 4) (Table 2) which led to the refinement of one question. Six focus groups of between two and six participants were conducted in meeting rooms in the hospitals or primary healthcare units respectively. The focus group with two participants was a small group [52] but data were retained for analysis because rejection would have wasted the participants’ time and the rich data. The focus groups lasted between 50 and 60 minutes, were audio recorded and transcribed verbatim. The quotes included in this article have been translated from Finnish to English and are used to demonstrate the credibility of the descriptions.

**Table 2:** Issues in the interview guide.

The interview guide focused in the following issues:

• Perceptions of the concept of collaboration. Example question: How do you define the concept of collaboration?
• Perceptions of nurse-to-nurse collaboration between hospital and primary healthcare nurses in older people nursing care. Example question: Which factors lead to good nurse-to-nurse collaboration between hospital and primary healthcare nurses in older people nursing care?
• Perceptions of preconditions and attributes of nurse-to-nurse collaboration between hospital and primary healthcare nurses in older people nursing care. Example question: What are the necessary preconditions of nurse-to-nurse collaboration between hospital and primary healthcare nurses in older people nursing care?
• Perceptions of the consequences of nurse-to-nurse collaboration between hospital and primary healthcare nurses in older people nursing care. Perceptions of how nurse-to-nurse collaboration may be developed between hospital and primary healthcare nurses in older people nursing care. Example question: How would you develop nurse-to-nurse collaboration between hospital and primary healthcare nurses in older people nursing care?

### Ethical considerations

The study followed national ethical standards [54]. Ethical approval was obtained from the Ethics Committee of the University (study number 28/2012) and approved by the participating organizations. All the participants received written and verbal information about the study and gave their informed consent voluntarily. Participants were told that they could withdraw from the study at any stage, that their anonymity would be protected and that data would be stored appropriately, during and after the study.

### Data analysis

Data were analyzed using dimensional analysis [50]. Dimensional analysis [50] is an alternative method for producing the basis of a substantive grounded theory about the social processes and the meanings of interactions in situations under scrutiny. The researcher kept memos during the data analysis [50] and used dimensionalization, differentiation and integration [50] to complete the analysis. After the second focus group, data were analyzed and compared to previously analyzed data in line with theoretical sampling techniques [49]. Data were collected, analyzed and compared until a “critical mass” of data and theoretical saturation in the analysis was achieved [50]. In the dimensionalization phase the transcripts were coded, and then combined into concepts and dimensions using constant comparison. This process facilitated a description of the properties of the data and the relationships between them forming the basis of a substantive grounded theory [50].

In the differentiation phase, an explanatory matrix was used as a framework to organize the data into a logical structure providing meaning to the phenomena [50]. This explanatory matrix identified the four conceptual components: the Context, Conditions, Processes and Consequences. In the integration phase data analysis was completed and the dimensions used to form a model of nurse-to-nurse collaboration between hospital and primary healthcare nurses in the older people care chain. After the analysis, the different phases of the dimensional analysis and the findings were discussed with all the authors and a consensus about how the data were theoretically interpreted was reached.

### Rigor

The rigor of the study is demonstrated through credibility, auditability and fittingness in the study [55]. The credibility, auditability and fittingness of the study was increased in six ways, 1) Through providing explanations and justifying the selection of the study participants, the sample, the setting and the theory generation method. 2) Using previous literature to select the sample size (at least 20 participants) [53] and the number of focus groups (6, n = 28) based on the likelihood of achieving a critical mass of data [53]. 3) *In vivo* coding [49,55] was used for the initial coding, to reduce the level of interpretation. 4) In a process of member checking [53] the researchers’ interpretation of the data were confirmed by half of the participants. 5) The interview guide was pilot tested (n = 4). 6) The analysis was supported throughout by the use of memos [50].

## Findings

**Figure 1:**
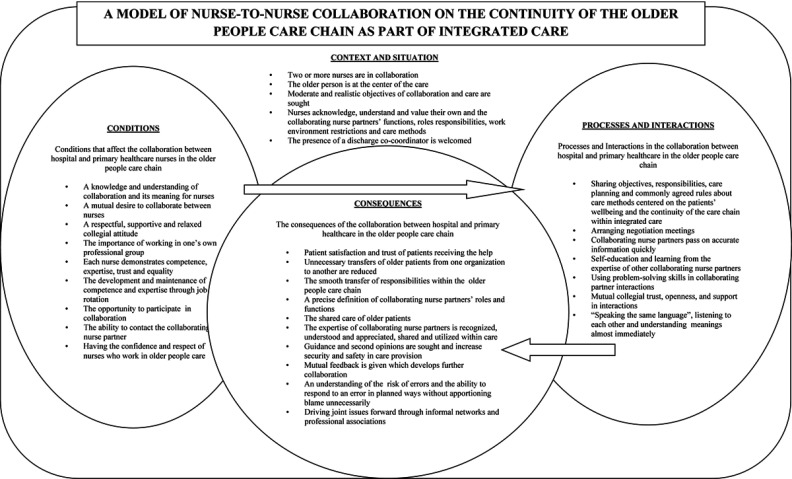
A model of the nurse-to-nurse collaboration on continuity of older people care chain as part of the integrated care from perspectives of hospital and primary healthcare nurses.

The analysis suggests that nurse-to-nurse collaboration in the older person care chain contains four dimensions. These dimensions are: 1) The Context and Situation, 2) The Conditions that affect nurse-to-nurse collaboration, 3) The Processes and Interactions within nurse-to-nurse collaboration and 4) The Consequences of nurse-to-nurse collaboration. These four dimensions form the basis of a model of nurse-to-nurse collaboration between hospital and primary healthcare nurses (Figure 1) as the basis of a substantive grounded theory.

### The Context and Situation of nurse-to-nurse collaboration

The participants perceived that nurse-to-nurse collaboration occurs in contexts where the functional capacity and individual care needs of older people are assessed. The collaborative assessment process, set up with the older person at the center of care, encouraged self-determination whilst ensuring that individual physical, medical and social needs were met.

“...we must see him as a whole, we must see that he is coping in the home and so that he has all the nutrition and medications, and the exercises and all these things in order in the home”. (Interview 4)

Changes caused by the aging process, including functional and mental capacity changes, were taken into account when setting objectives after assessment. This study suggests that nurse-to-nurse collaboration works to moderate these objectives and ensured that expectations were realistic.

When two or more nurses are in collaboration they are described as collaborating nurse partners. Knowing and meeting a collaborating nurse partner was found to be an essential part of effective collaboration. During the collaboration process nurses acknowledged, understood, and valued their own and collaborating nurse partners’, functions, roles, responsibilities, work environment restrictions and care methods. This acknowledgement brought smoothness, facilitated planning, avoided false promises and increased understanding about care methods. In this context nurses increased the sense of security and trust that older people perceived in their care.

“That we know little what the other organization does... we definitely need more [information]. We should know accurately in here how primary healthcare works and what do they offer to these patients, so that we can give proper instructions to the patient and the other way around”. (Interview 6)

We found that discharge coordinators were useful because they supported collaborating nurses and facilitated the safe, smooth transition between hospital and primary healthcare and *vice versa*. This coordination role required sufficient knowledge of both organizations for example, who to contact and how they operated.

### Conditions that affect nurse-to-nurse collaboration

The Conditions are those factors that affect the Processes and Interactions during collaboration. This study suggests that the Conditions affect how well collaboration works through the provision of opportunities to collaborate, a knowledge and understanding of the meaning of collaboration and how this can be used to improve older people nursing care. From a process perspective, important factors were the nurses’ mutual desire to collaborate with a respectful, supportive and relaxed collegial attitude toward collaborating nurse partners and a positive attitude toward the construction of collaborating partnerships and networks. As one nurse said:

“[Collaboration requires a] Respect for one another so that one is not less important than another whether you are working in a nursing home or outpatient clinic or anywhere else”. (Interview 5)

There were some relationship factors found that affected collaboration. Nurses in collaboration needed to demonstrate equality, trust, collegiality and a personal interest in their collaborating nurse partners, their work and the success of the collaboration. These relationship factors facilitated the development of professional networks within and between organizations, through discussion about relevant nursing issues. Closely associated with this networking was the development and maintenance of competence and expertise which nurses in this study developed through their collaboration. Mutual consultation and education in collaboration helped to construct useful networks providing opportunities to develop new ways of working through the management of common problems in patient care. Related to personal development, healthcare organizations usually provide education opportunities such as job rotation. In this study, these experiences were found to be valuable and were used to develop competence and new understandings of care delivery further improving collaboration.

“I should be able to rely on the professional skills that are in primary healthcare, the skills of the nurses there. In a way… in a way that in here you can feel confident to send a patient there for further care and know that he will be well cared [for] there”. (Interview 3)

The provision of up-to-date information about the function of the collaborating organization from their web sites, personal contact information and nurses’ availability were also found to facilitate timely communication and smoothing of the care chain.

“Still, I think that collaboration could be even better. It feels somehow in a way that there is not enough amount of time to share the mutual information”. (Interview 6)

We found that expressions of confidence and respect had an essential role creating favorable conditions for nurse-to-nurse collaboration within older people care. Managers seem to have a role in this by encouraging collaborating partners to work in a relaxed manner.

### Processes and Interactions in nurse-to-nurse collaboration

The Processes and Interactions which occur when nurses collaborate have been reported to be shaped by the existing Conditions [50]. In this study the Processes and Interactions were: shared objectives; care planning and commonly agreed rules about care methods centered on the patients’ wellbeing and the continuity of the care chain.

“[It is important that collaborating nurses]…have a common objective and goal. Everyone will do the work in different organizations [differently] but collaborate with each other”. (Iinterview 5)

We found that the interests of their patients were central to collaborating nurse partners who worked actively and courageously to make sure their patients were heard and their needs met during discharge from hospital. In this transition, collaborating nurses advocated for their patients for example, with regard to: care expenses, safety and by providing comprehensive verbal and written instructions for home care workers and patients’ relatives. We found that collaborating nurse partners working in this complex area of health care had to be flexible in their approach to negotiation and care planning to ensure a smooth transition between hospital and primary healthcare. The negotiations were centered on the older people, their care processes, care methods and the documentation of these throughout the care chain. In practice, this meant that nurses arranged negotiation meetings with all relevant parties, sometimes in the patient’s own home. At these meetings the goals of care, the shared responsibilities and the management of care policies, the care methods and care instructions were discussed to ensure the continuity of patient care.

“To sit around a table and think together specifically about the older patient how to manage the chain of care [so transition] could be seamless and improved”. (Interview 4)

To ensure the success of care in the older people care chain, collaborating nurse partners passed on accurate information quickly from one organization to another. This transfer of information was particularly important in cases where there was key information that had to be delivered accurately, timely and appropriately by nurse partners for example, about medication management. To help with this transfer of information nurses required access to effective electronic systems of communication for use by all the collaborating partners. When nurses acted promptly and collaboratively to create an accurate and useful record of up-to-date information about the older patient, optimal care was more likely to be achieved.

“That’s why we do this work [so] that the chain of care doesn’t break in information transfer and the patient receives the best possible care, regardless of where the patient is taken care of at the moment”. (Interview 4)

Collaborating nurse partners also learned from each other. This included learning related to the care provided to the older person and the workplace for example, the use of guidelines.

“We share a lot of our special expertise with each other, because we all have maybe a little bit [of our] own expertise [and] areas which we do know more and that others do not know”. (Interview 6)

We found that in difficult situations, nurses supported each other using problem-solving skills within collaborative interactions. To achieve this and be effective, interactions occurred within a friendly atmosphere filled with mutual trust, openness and support. Under these circumstances nurses were more likely to be able to converse easily using the same terms and inferences, that is “speak the same language”, listen to each other and understand meanings almost immediately.

### Consequences of the nurse-to-nurse collaboration

The Consequences describe the elements of nurse-to-nurse collaboration which are determined by the Processes and Interactions [50]. We found that good nurse-to-nurse collaboration led to patient satisfaction and improved trust as patients received the help they needed from the collaborating nurse partners. Additionally, the participants perceived that good collaboration led to the prevention of unnecessary transfers of older patients from one organization to another. These Consequences of collaboration were also thought to affect the older person’s care more strategically through the development of trusted and successful care processes. The participants perceived that these trusted processes could develop into better interaction channels and new guidelines about for example, hospital and primary healthcare nurses’ joint home visits.

“It is probably also related to trust, because if the patient is satisfied, then he better trust that the healthcare is working properly in Finland. He has received all the help that he needs without having to be transferred here and there and lined up here and there”. (Interview 4)

Another consequence of good nurse-to-nurse collaboration was found to be the smooth transfer of responsibilities between nurses in hospital and primary healthcare organizations. A smooth transfer occurred when the collaborating nurse partners’ roles and functions were defined precisely taking into account their specialized and broader expertise appropriately. Useful nurse-to-nurse collaboration occurred when the expertise of the collaborating nurses was recognized, understood and appreciated, shared and utilized within care. In these circumstances, it was perceived that collaboration could provide opportunities for second opinions and guidance about nursing care decisions leading to increased security and safety in the care provision.

“Also collegial, because you can always ask and make sure from the other if you are bit unsure or don’t know or you would like a second opinion of the situation”. (Interview 4)

Collaborating nurse partners provided feedback to each other which developed further collaboration. We found that it was important that this feedback was delivered in mutual dependence, included an understanding of the risk of errors and was supportive, for example error responses did not apportion blame unnecessarily. A Consequence of good nurse-to-nurse collaboration feedback was network development between organizations. This networking became stronger as collaborating partners drove joint issues forward through informal networks and professional associations.

“The network mapping takes quite a long time, but it is very useful and does help with collaboration [making sure that] in general all the matters are dealt with”. (Interview 1)

## Discussion

Grounded theory is a suitable methodology to develop a substantive theory around the phenomena under scrutiny [51]. An earlier study reported that collaboration requires healthcare managers to provide opportunities for meetings, the development of relationships, effective communication and trust [24]. The model presented in this study supports these findings and goes further identifying the elements essential for nurse-to-nurse collaboration between hospital and primary healthcare nurses within the older people care chain. Knowledge of these elements is important if nurse-to-nurse collaboration is to be improved in these complex environments [3]. It seems that better collaboration will improve the care of older people who require a seamless service between organizations [32,34]. This knowledge can be used to support the ongoing care reform and integration process in Finland, helping nurses who care for the older people to improve the continuity of services. The results may also be useful in other countries.

This study found that nurse-to-nurse collaboration between hospital and primary healthcare nurses within the older people care chain, is an essential part of a nurses’ work role. This collaboration can be divided into to four dimensions: Context and Situation, Conditions, Processes and Interactions and Consequences, which has been conceptualized in a model of nurse-to-nurse collaboration (Figure 1). Considering the Context and Situation, when nurse-to-nurse collaboration occurred with the older person at the center of care, the expectations of care throughout the older people care chain were moderated and made more realistic. Nurses in collaboration developed new understandings, acknowledged and valued their own and collaborating nurse partners’ functions, roles, responsibilities, work environment restrictions and care methods. This awareness has been highlighted as useful in previous studies [37,41,46,47]. In this current study we found that in the Context and Situation it was useful to use coordinators who worked between hospital and primary healthcare to support the collaborating nurses. This finding supports previous studies reporting that collaboration and the continuity of care can be improved by using liaison nurses to coordinate and organize discharge planning and the communication between hospital and primary healthcare organizations [43,44,45].

The findings of this study suggest that the Conditions are the power behind the meaning of collaboration for nurses and provide opportunities to improve Processes and Interactions in nurse-to-nurse collaboration. This finding is supported by Kools et al*.* [50] who reported that the Conditions have an important impact shaping actions and interactions. Previous literature has reported that the interaction in collaboration should include respect [35,46], trust and openness [46]. The findings of this study support this and provide more detail about the issues. Conditions in collaboration included the atmosphere in which the work takes place. Good collaboration required a mutual respect for nurses and patients and an environment which is supportive, facilitating and provides opportunities for collaborating partners to work with a relaxed attitude. Supporting earlier literature [23], the Conditions also included the opportunity to work in one’s own professional group and own area of expertise and responsibility. We found that nurses worked to increase their own knowledge in specific areas and were active in developing their expertise. This self-educative aspect of a nurse’s work was helped by the opportunity to participate, organize and consult within collaboration which occurred when collaborating nurses were able to contact each other easily. Opportunities to participate, consult and collaborate were perceived to be facilitated by job rotation. Kirsebom et al. [32] also recommended job rotation between different organizations as a means of improving collaboration.

Nurses shared objectives, planned care and agreed common rules and care methods within the collaboration process. This finding is supported by previous studies reporting that collaboration is enhanced by: mutual dependency; shared decision-making; a common health care philosophy and values; pooled information and care planning [22]; shared objectives [38] and appropriate meetings with collaborating partners [37,46]. Expanding this notion of appropriate meetings with collaborating partners, we found that collaboration was enhanced when partners were able to make timely contact and maintain this contact to transfer and share current and accurate information. This development is given credence by a previous study which reported that hospital and primary healthcare nurses cited poor information exchanges as a barrier to collaboration when older patients were transferred between settings [33], Education has also been found to promote collaboration [37,46]. In this study, conceptualized within Processes and Interactions, nurses were found to learn from each other as they shared their expertise and developed the expertise of their collaborating partners during the collaboration process.

Nurses require several skills [36,37] in collaboration for example, decision-making, problem-solving, leadership and conflict management [36,47]. However, nurses must also acknowledge their own humanity and limitations [38]. This current study supports these previous studies in terms of the skills required for collaboration. However, the need for the acknowledgement of personal humanity and limitations was not found. In this study, “speaking same language” in the interaction was found to be important for good collaboration.

The last category in the model is the Consequences of nurse-to-nurse collaboration which we suggest leads to older patients’ satisfaction and trust in their healthcare. Good nurse-to-nurse collaboration also leads to the smooth transfer of responsibilities, a more precise definition of the functions of the collaborating nurses and the shared care of older people. These elements of nurse-to-nurse collaboration seem to operate synergistically to reduce the number of unnecessary transfers of older people between organizations.

Another Consequence of collaboration we found was an awareness of nurses’ competencies and the use and sharing of that competence in collaboration. One of those competencies was the ability to provide feedback to the collaborating partners. Feedback from successful collaboration was provided as a Consequence of collaboration leading to an awareness of risk and how to respond to error events in planned ways without apportioning blame unnecessarily. Also collaborating nurses jointly drove forward common issues through informal networks and professional associations. The Consequences of nurse-to-nurse collaboration have been neglected in previous literature.

There are millions of nurses [11] working in collaboration to ensure the integration and continuity of care within the care chain. In that chain, healthcare systems need to respond to increasingly complex situations [3]. Worldwide, hospital and primary healthcare has moved from being based within a hierarchy towards a network of collaborative service provision [1]. Within these networks, nurses have an important role [27] in ensuring seamless health services for older people [3]. Currently, there is lack of research regarding collaboration between nurses [40] and between hospital and primary healthcare organizations [35,41]. The findings of previous studies about collaboration have focused on elements that in this study were conceptualized within Processes and Interactions. Kools et al. [50] reported that the Conditions are the most salient elements of social processes because they have an influence on the other elements. This current study suggests that research effort on collaboration should focus on understanding how Conditions affect the Processes and Interactions in nurse-to-nurse collaboration between hospital and primary healthcare nurses within the older people care chain.

### Limitations

Some limitations need to be taken into account in the interpretation of the findings of this study. The data were taken from nurses in one hospital district in Southern Finland. Hospital and primary healthcare units within this district were selected because they were charged with caring for older people. However, older people use different services and the findings may have been different in other study settings completed at different times. The method used in the study was appropriate but data from the six focus groups were not fully analyzed before the next interview was conducted as this was precluded by the schedule of interviews. However, the completed analysis demonstrated that this did not affect the qualitative direction of the focus groups.

This study provides the basis of a substantive theory about the structure of nurse-to-nurse collaboration between hospital and primary healthcare nurses within the older people care chain from the perspectives of a sample of Finnish hospital and primary healthcare nurses. The results should be considered preliminary as there may be more elements of nurse-to-nurse collaboration than those identified. In Finland, the provision of healthcare services is managed using nationally agreed care chain policies which may limit the use of the results outside Finland. In other contexts and countries, nurses follow other care chain policies which may make collaboration different in those countries. However, in Finland the care chains are centered on similar evidence-based clinical practice guidelines as produced in many other countries, suggesting the results may be more widely usable. The complexities of the phenomena require more studies to test the model empirically to determine the generalizability of the findings and to see if the model can be used predicatively in various contexts, considered from different perspectives for example, those of older people.

## Conclusion

Health services are provided in multiple settings requiring health professionals to collaborate. Collaboration within the older people care chain between hospital and primary healthcare seems to be important to the integration of services and the continuity of care. Although effective collaboration is necessary, collaboration could be improved markedly within current healthcare systems. Our findings shed light on the nature of nurse-to-nurse collaboration suggesting that it is a complex phenomenon which can be conceptualized in a preliminary framework containing four dimensions. An in-depth understanding of the collaboration process using these dimensions provides direction and opportunities to develop nurse-to-nurse collaboration systematically.

These findings could be used to support and strengthen healthcare services developing a seamless care chain for older people. This strengthening could include the planning and implementation of new collaboration policies for work between hospital and primary health care organizations. This development will promote the safe, timely and controlled transfer of older patients between organizations enhancing the continuity of care for those patients. Strengthening nurse-to-nurse collaboration could have a positive effect on whole teams of healthcare professionals. However, the Conditions in which collaboration takes place seem to be important. The Conditions include the development of collaboration expertise in mutual, collegial, trusting, open, relaxed and supportive interactions with shared objectives and commonly agreed rules and care methods. These findings can be also used in nurse education to provide guidelines for the enhancement of the skills required in nurse-to-nurse collaboration in the older people care chain as part of the integrated care.
